# Effect of Green Tea Extract on Doxorubicin Induced Cardiovascular Abnormalities: Antioxidant Action

**Published:** 2011

**Authors:** Leena Patil, R Balaraman

**Affiliations:** a *Dayananda Sagar College of Pharmacy, Shavige Malleshwara Hills, Kumarswamy Layout, Bangalore-560 078, Karnataka, India*.; b *Pharmacy Department, Faculty of Technology and Engineering, The M.S. University of Baroda, Baroda-390 001, Gujarat, India.*

**Keywords:** Doxorubicin, Green tea, Antioxidant, Catechins, Electrocardiogram, Blood pressure

## Abstract

Doxorubicin (DOX) induces oxidative stress leading to cardiovascular abnormalities. Green tea extract (GTE) is reported to possess antioxidant activity mainly by means of its polyphenolic constituent, catechins. Our study was aimed to find out the effect of GTE (100 mg/kg / day p.o. for 28 days) on DOX induced (3 mg/kg, IP on days 1, 7, 14, 21, 28) cardiovascular abnormalities in rat heart. DOX treatment led to significant increase in blood pressure, ST interval, serum levels of LDH, CK, SGOT, lipid peroxidation .The antioxidant enzymes such as super oxide dismutase, catalase and reduced-glutathione were decreased considerably in the heart of DOX treated rats as compared to the normal control. A combined treatment with GTE and DOX showed a considerable decrease in serum markers of cardiotoxicity such as LDH, CK, SGOT and lipid peroxides. There was significant increase in the activities of antioxidant enzymes and also showed improvement in hemodynamic parameters and ECG changes as compared to DOX treated animals. DOX treatment caused disorganization of myocardial tissue which was restored in animals treated with GTE along with DOX. Thus it can be concluded that GTE possesses an antioxidant activity and by virtue of this action it can protect the heart from DOX induced cardiovascular abnormalities.

## Introduction

It has been widely reported that doxorubicin, an anthracycline antibiotic for cancer treatment, causes cardiotoxicity due to the production of free radicals (1-4). The clinical effectiveness of doxorubicin treatment for several cancers is affected by the dose-limiting side effect cardiotoxicity (5). In the past, several studies have concluded that antioxidants like *α*-tocopherol (*α*TC) (6), a-phenyl-tert-butyl-nitrone afforded protection from doxorubicin induced myocardial injury without affecting its antineoplastic activity (7). 

Polyphenols are plant metabolites occurring widely in plant food and possess outstanding antioxidant and free radical scavenging properties (8-9). Green tea is an excellent source of polyphenolic antioxidants, particularly of a group known as green tea catechins (GTCs) (10). Green tea reduces iron-induced lipid peroxidation in brain homogenates as well as in cultured C_6_ astrocytes and lung cells (11-12). In addition, green tea has been shown to reduce the formation of the spin-adducts of hydroxyl radicals and hydroxyl radical-induced DNA strand breakage *in-vitro *(13). Green tea has been found to have inhibitory effects on the chemical-induced lung tumorigenesis (14). There is also considerable epidemiological evidence suggesting that the consumption of green tea lowers the risk of heart disease as well as several types of cancer incidences as a result of these antioxidant mechanisms (15). 

However, to the best of our knowledge, effect of GTE on doxorubicin induced cardiovascular abnormalities in rat heart has not been explored yet. Therefore, the aim of present study was to evaluate the protective effect of GTE against DOX induced cardiovascular abnormalities through serologic analyses, biochemical analyses, hemodynamic changes and histopathology study.

## Experimental

*Chemicals*

Standardized powdered, ethyl acetate extract of green tea leaves (c*amellia sinensis*) was obtained as a gift sample from Cherain Chemicals, Baroda, India with total polyphenolic content 35%. Doxorubicin injection was obtained as a gift sample from serum institute of india Ltd., Pune. Super oxide dismutase, malondialdehyde, catalase standards were purchased from sigma Aldrich; USA. Reduced glutathione, 5, 5’Dithiobis (-2 nitrobenzoic acid), thiobarbituric acid from Hi Media; India. All other chemicals were of analytical grade.

*Animals*

Adult albino rats of either sex (wistar strain) weighing between 200 and 250 g were used for the study. The animals were fed ad libitum with standard pellet diet and had free access to water. All experiments and protocols described in the present study were approved by the institutional animal ethics committee (IAEC) of M. S. University, Baroda and are in accordance with guidelines as in “Guide for the care and use of laboratory animals” published by NIH publication (NO 85-23 revised 1996) and with permission from Committee for the purpose of control and supervision of experiments on animals (CPCSEA), Ministry of Social Justice and Empowerment, Government of India.

*Experimental protocol*

*Chemical analysis of green tea extract*

TLC fingerprint profile of the extract was established using HPTLC. For the development of TLC fingerprint, 500 mg of the powdered green tea extract was extracted with (3x25 mL) of methanol. The extracts were pooled, filtered and concentrated to 25 mL. Suitably diluted stock solution of methanolic extract with gallic acid standard solution and catechin were spotted on a pre-coated Silica gel G60 F254 TLC plate (E.Merck) using CAMAG Linomat IV Automatic Sample Spotter and the plate was developed in the solvent system of Toluene: Ethyl acetate: Formic acid (6 : 6 : 1). The plate was dried at room temperature and scanned using CAMAG TLC Scanner 3 at UV 254 nm and r_f_-values, and peak area of the resolved bands were recorded. Relative percentage area of each band was calculated from peak areas. The TLC plate was derivatised by spraying with 5% methanolic ferric chloride solution for the detection of phenolic compounds.

*Groups and treatment schedule*

Powdered green tea extract was reconstituted in distilled water. Doxorubicin injection was dissolved in sterile water for injection. The animals were divided into four groups each consisting of six rats and received the following treatment

• Group I (control): Received distilled water (3 mL/kg /day p.o. for 28 days) and sterile water for injection (1 mL/kg, IP) on day 1, 7, 14, 21, 28. 

• Group II (DOX): Doxorubicin injection (3 mg/kg IP) on day 1, 7, 14, 21, 28. 

• Group III (DOX + GTE): Green tea extract (100 mg/kg /day p.o. for 28 days) and doxorubicin injection (3 mg/kg IP) on day 1, 7,14,21,28.

• Group IV (GTE): Green tea extract (100 mg/kg /day p.o.) for 28 days.

*Hemodynamic study*

Blood pressure was measured non invasively at the start of study and at weekly intervals by tail cuff method using LE 5002 storage pressure meter (LETICA scientific instruments, SPAIN) in all the above mentioned groups. For the blood pressure measurements animals were trained for at least 1 week until blood pressure was steadily recorded with minimal stress and restraint. The mean of 10 measurements of trained animals was recorded.

*Electrocardiographic measurements*

After 48 h of the last injection of either doxorubicin or vehicle, ECG were recorded through needle electrodes (Lead II) using Biopac MP30 data acquisition system (Biopac Systems, Santa Barbara, CA). The changes in Heart rate, ST interval and QT interval were determined from ECG. 

*Serum markers*

After 48 h of the last injection of either doxorubicin or vehicle, Blood was collected by retro-orbital route for serological analyses. Serum levels of lactate dehydrogenase (LDH) and serum creatine kinase (CK) were determined by using standard kits of Reckon Diagnostic Ltd, India while glutamic oxaloacetate transaminase (SGOT) was estimated by using standard kit of Span Diagnostic Pvt Ltd, India. 

*Biomarkers of the oxidative stress*

After 48 h of the last injection of either doxorubicin or vehicle, the heart was excised under euthanasia in chilled tris buffer (10 mM pH 7.4) for measurement of tissue markers of oxidative stress. The excised heart was then weighed and homogenized in chilled Tris buffer (10 mM, pH 7.4) at a concentration of 10% (w/v). The homogenates were centrifuged at 10,000×*g *at 0^◦^C for 20 min using Remi C-24 high speed cooling centrifuge. The clear supernatant was used for the assays of malondialdehyde content as indicator of lipid peroxidation (LP)(16), endogenous antioxidant enzymes, superoxide dismutase (SOD) (17), catalase (CAT) (18), reduced glutathione (GSH) (19) and total proteins (20). 

*Histopathologic examination*

For histotological evaluation, the specimens were fixed in 10% formalin, dehydrated and embedded in paraffin. Tissues were then sectioned at 4 μm, stained with haematoxylin and eosin (H&E) and examined for histopathological evidence under Olympus BX40 Photomicroscope.

*Statistical analysis*

Results of all the above estimations have been indicated in terms of mean ± SEM Difference between the groups was statistically determined by analysis of variance (ANOVA) followed by Tukey-Kramer multiple Comparisons test with the level of significance set at p *≤ 0.05.*

## Results

*Chemical analysis*

**Figure 1:**
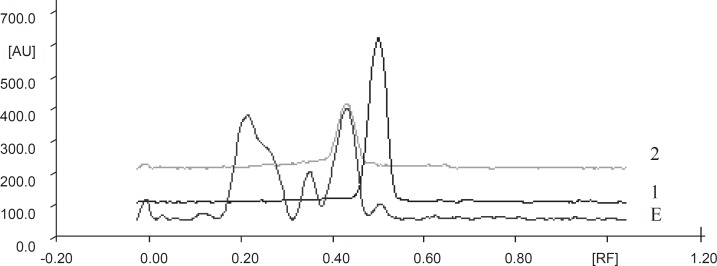
TLC densitometric chromatogram of methanolic extract of green tea with gallic acid standard and catechin standard solution. E: Extract, 1: Gallic acid, 2 : Catechin standard solution

The fingerprint chromatograms are shown in Figure 1. Details of the fingerprint analysis are given in Table 1. Catechin content of extract was analyzed using ethyl acetate as medium and found that it contains 35% catechins.

**Table 1:** Details of fingerprint chromatograms of GTE after scanning at 254 nm

**Extract**	**Solvent system**					**No. of spots**		
Methanolic extract	Toluene: Ethyl acetate: Formic acid (6 : 6 : 1).					8		
r_f_ -values	0.03	0.12	0.22	0.35	0.43	0.50	0.63	0.68
Relative %	3.30	1.84	33.03	15.11	35.09	4.99	1.27	1.05

*Electrocardiographic changes*

There was a significant increase in ST interval as well as QT interval while heart rate was significantly decreased in DOX treated rats as compared to the control rats. GTE treatment for 28 days along with DOX significantly restores ECG changes towards normalcy (Table 2). 

**Table 2:** Effect of administration of DOX alone and along with GTE on ECG

**Groups**	**ST interval (msec)**	**QT interval (msec)**	**Heart rate (bpm)**
**Control (Group I)**	29.16 ± 1.53	62.5 ± 1.11	403.66 ± 9.51
**DOX (Group II)**	62.5 ± 2.14^***^	96.66 ± 3.8^***^	280.83 ± 23.28^***^
**DOX+GTE (Group III)**	33.33 ± 2.78^***^	67.5 ± 2.81^***^	395.83 ± 4.72^***^
**GTE (Group IV)**	30.0 ± 1.29	66.07 ± 2.06	389 ± 10.21
**f-value**	62.27	35.81	17.71
**p-value**	p < 0.0001	p < 0.0001	p < 0.0001

Values are expressed as mean ± SEM (n = 6), group II was compared with group I, group III was compared with group II, ^***^p < 0.001, NS = Non significant

*Serum markers*

DOX administration significantly increased serum level of CK, LDH and GOT as compared to control rats. The administration of GTE along with DOX significantly restored serum marker levels towards control value as compared to DOX alone group. There was no significant change in serum markers of GTE alone group (Table 3).

**Table 3:** Effect of administration of DOX alone and along with GTE on serum markers of cardiotoxicity

**Groups**	**Lactate Dehydrogenage (U/L)**	**Creatine Kinase (U/L)**	**SGOT (U/mL)**
**Control (Group I)**	169.83 ± 4.62	231.16 ± 12.68	32.33 ± 2.0
**DOX (Group II)**	610.33 ± 77.66 ^***^	511.5 ± 17.69 ^***^	102.05 ± 5.86^***^
**DOX+GTE (Group III)**	307.16 ± 18.04 ^***^	261.66 ± 17.77^***^	41.94 ± 2.35^***^
**GTE (Group IV)**	177.5 ± 6.02	240 ± 16.32	31.33 ± 2.4
**f-value**	26.45	68.19	91.68
**p-value **	< 0.0001	< 0.0001	< 0.0001

Values are expressed as mean ± SEM (n = 6), group II was compared with group I. group III was compared with group II. ^***^p < 0.001, NS = Non significant

*Biomarkers of the oxidative stress*

There was a significant increase in LP and significant decrease in GSH, SOD and CAT levels in DOX treated rats as compared to control rats. The administration of GTE along with DOX significantly improves the levels of GSH, SOD and CAT and reduced LP as compared to DOX alone group. There was no significant change in SOD, CAT, GSH and LP of GTE alone group (Table 4).

**Table 4:** Effect of administration of DOX alone and along with GTE on biomarkers of the oxidative stress

**Groups **	**Lipid peroxidation ** **(nmoles of MDA / mg protein) **	**Reduced glutathione ** **(μg ofGSH/ mg protein) **	**Superoxide dismutase (Units/mg protein) **	**Catalase ** **(μmoles of H**2**O**2 **consumed / ** **min/mg protein) **
**Control (Group I) **	3.06 ± 0.16	9.45 ± 1.21	2.33 ± 0.36	4.02 ± 0.32
**DOX (Group II) **	4.75 ± 0.28^***^	5.14 ± 0.15^***^	0.6 ± 0.18^** ^	1.85 ± 0.18^*** ^
**DOX+GTE (Group III) **	2.98 ± 0.06^*** ^	8.40 ± 0.23^** ^	2.15 ± 0.27^** ^	4.61 ± 0.29^*** ^
**G TE (Group IV) **	2.75 ± 0.20	8.87 ± 0.59	2.16 ± 0.33	4.41 ± 0.47
**f-value **	21.89	7.77	7.33	14.24
**p-value **	p < 0.0001	p = 0.0012	p = 0.0016	p < 0.0001

Values are expressed as mean ± SEM (n = 6), group II was compared with group I, group III was compared with group II, ^**^p < 0.01, ^***^p < 0.001, NS = Non significant

*Hemodynamic study*

**Figure 2:**
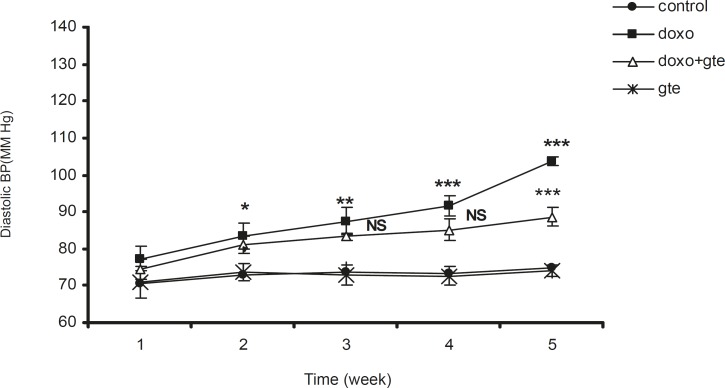
Effect of administration of DOX alone and along with GTE on diastolic blood pressure. Values are expressed as mean ± SEM (n = 6). Group II was compared with group I. Group III was compared with group II. ^*^p < 0.05, ^**^p < 0.01, ^***^p < 0.001, NS = Non significant

There was a significant increase in systolic, diastolic and mean blood pressure in DOX treated group as compared to control group. GTE treatment for 28 days along with DOX significantly reduced the blood pressure normal as compared to DOX alone group (Figure 2-Figure 4 ). There was no significant change in systolic, diastolic, mean blood pressure and heart rate of GTE alone group.

**Figure 3:**
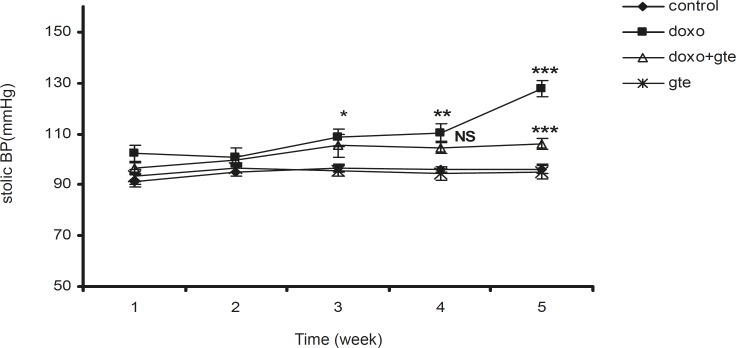
Effect of administration of DOX alone and along with GTE on systolic blood pressure. Values are expressed as mean ± SEM (n = 6). Group II was compared with group I. Group III was compared with group II. ^*^p < 0.05, ^**^p < 0.01, ^***^p < 0.001, NS = Non significant

**Figure 4:**
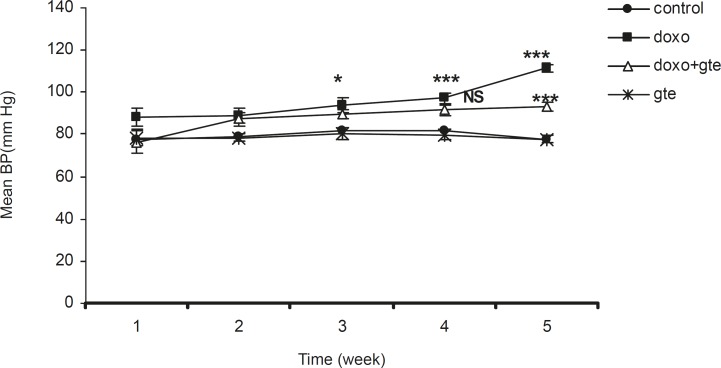
Effect of administration of DOX alone and along with GTE on mean blood pressure. Values are expressed as mean ± SEM (n = 6). Group II was compared with group I. Group III was compared with group II. ^*^p < 0.05, ^**^p < 0.01, ^***^p < 0.001, NS = Non significant

*Histopathologic examination*

**Figure 5:**
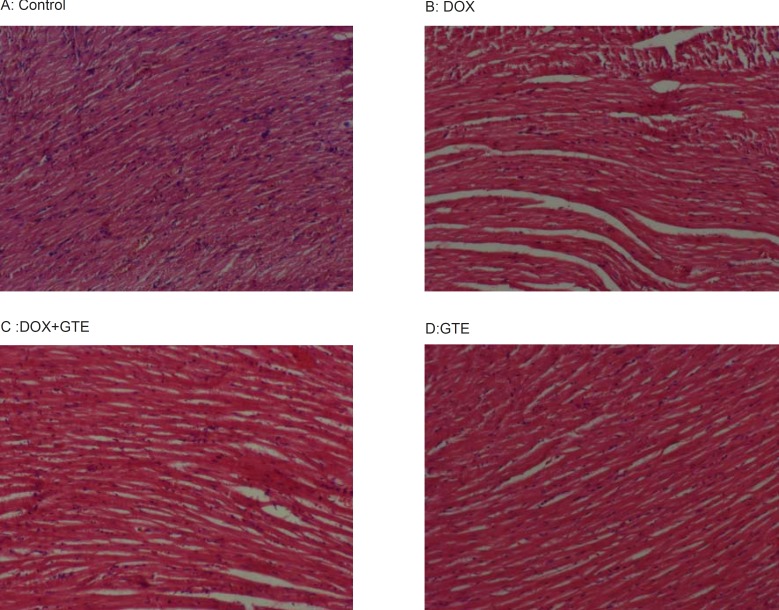
Cross sections of hearts in rats treated with DOX and along with GTE. hearts from control (A) and GTE treated rats (D) Shows normal feature of myocardium. However, hearts from a doxorubicin treated rats (B) Shows a massive necrosis of heart muscle fibres along with focal loss, marked fragmentation and disorganization of myocardial fibres. Administration of GTE along with DOX (C) Restored these changes towards normalcy

DOX treated animal shows a massive necrosis of heart muscle fibres along with focal loss and marked fragmentation. Disorganized arrangement with no well-defined boundaries or distinct bundles of myocardial fibers was observed. Nuclei were scattered, some were lost and some were picnotic in nature. Administration of GTE along with DOX restored these changes towards normalcy (Figure 5).

## Discussion

The involvement of free radicals in the mechanism of doxorubicin induced cardiotoxicity has been the subject of a number of reviews (21-23). In the present, the results indicate that study intraperitoneal administration of DOX at a total dose of 15 mg/kg for 5 week induces cardiovascular abnormalities by increase in free radical production as indicated by significant increase in LDH, CK, SGOT and lipid peroxidation. These results are consistent with earlier studies (21). Administration of GTE along with DOX causes significant decrease in LDH, CK, SGOT and lipid peroxidation near to that of control group. These data suggest that GTE may protect the myocardial tissue against DOX induced cardiotoxicity. It was reported that DOX affects the hemodynamic parameters (24). In our study, DOX treatment causes a gradual increase in systolic, diastolic and mean blood pressure as compared to control rats. GTE administration along with DOX restores blood pressure towards normal value. These results are consistent with the earlier studies (25-27) where it was hypothesized that the increase in BP may be due to catecholamine release. The results of present study clearly demonstrated that there is an increase in myocardial injury as indicated by increase in ST interval and QT interval and a decrease in heart rate of ECG pattern in DOX treated group. Administration of GTE along with DOX restores ECG changes towards normalcy. 

Further results also led to the belief that administration of GTE improves the biochemical marker levels indicating a decrease in oxidative stress as evident by increased levels of GSH, SOD and CAT with decreased production of LP. These protective effects are also supported by the restoration of serum marker enzymes, EGC changes and histopathology study. It seems that antioxidant agents can protect the heart from doxorubicin induced assault as confirmed by several studies and reviewed (28, 29). It has been reported that catechins are important constituents of green tea that are responsible for antioxidant and protective effects. We verified the catechin content of the extract using ethyl acetate as medium and found that it contains 35% catechins. Furthermore, it was also reported that GTE exhibits more potent antioxidant activity than other conventional antioxidants like vitamin E and C in addition to its anti cancer action (15). 

As a result, GTE could be a better option for ameliorating doxorubicin induced cardiotoxicity. We conclude that the GTE is able to prevent the cardiovascular abnormalities and pathological changes in biochemical markers, which were induced by doxorubicin. This protection may be due to the catechin content of GTE, which is found to be a potent antioxidant among many counterparts.
